# An AI-Based Radiomics Model Using MRI ADC Maps for Accurate Prediction of Advanced Prostate Cancer Progression

**DOI:** 10.3390/curroncol33010035

**Published:** 2026-01-08

**Authors:** Kexin Wang, Pengsheng Wu, Yuke Chen, Huihui Wang

**Affiliations:** 1 Department of Radiology, Peking University First Hospital, Beijing 100034, China; kexin_wang@mail.ccmu.edu.cn; 2 Beijing Smart Tree Medical Technology Co., Ltd., Beijing 102200, China; wupengsheng@smart-imaging.cn; 3 Department of Urology, Peking University First Hospital, Beijing 100034, China; 1861008064@pku.edu.cn

**Keywords:** prostate cancer, magnetic resonance imaging, apparent diffusion coefficient, radiomics, survival analysis

## Abstract

The use of deep learning radiomics to predict whether advanced prostate cancer (PCa) will progress within two years after treatment has been validated, yet there remains a lack of research on estimating time to progression. Patients were enrolled from October 2017 to March 2024. One hundred and eighty-two patients with advanced PCa diagnosed through ultrasound-guided systematic prostate biopsy were enrolled. A deep learning-based radiomics model for predicting progression was firstly developed using pretreatment MR apparent diffusion coefficient (ADC) maps, and the performance of manual (ROIref) versus AI-derived (ROIai) tumor segmentations was compared. Then, survival analysis was performed to compare ROIref-based and ROIai-based radiomics-predicted probabilities in the risk stratification. The area under the receiver operating characteristics curve (AUC) was used to estimate the model efficacy. The model achieved high AUC values for progression prediction in test sets (ROIref: 0.840, ROIai: 0.852). No significant difference was observed between ROIai-based and ROIref-based approaches (ΔAUC = 0.012, *p* = 0.870) in the test set. Both ROIref-predicted and ROIai-predicted probabilities independently predicted progression in multivariate Cox proportional hazard regression models (*p* < 0.001) and stratified patients into distinct survival groups (log-rank *p* < 0.001). Decision curve analysis confirmed equivalent clinical utility across thresholds (0.1–0.6), with net benefit exceeding the “treat all” and “treat none” strategies. In conclusion, deep learning-based radiomics models could effectively predict advanced PCa progression, with AI-derived tumor annotations performing equally to manual expert ones.

## Simple Summary

Advanced prostate cancer (PCa) is prone to recurrence and metastasis after treatment. Previous studies have demonstrated the potential of deep learning radiomics to predict 24-month progression in advanced PCa using pretreatment MRI ADC map-derived features, outperforming both traditional radiomics and clinical models. In our study, we first conducted a comparison of manual and AI-generated segmentation impacts, demonstrating equivalent prognostic value and resolving prior concerns about annotation dependency. Then, we established a radiomics model as a time-to-event predictor, with stable discrimination (48-month AUC > 0.75) and calibration (Brier score < 0.15), enabling risk-adapted surveillance intervals—a capability absent in earlier models. These innovations collectively advance radiomics toward clinically actionable, observer-agnostic tools for precision oncology.

## 1. Introduction

Prostate cancer (PCa) ranks as the second most common cancer and the fifth leading cause of cancer mortality among men worldwide, with an estimated 1.4 million new cases and 375,000 deaths in 2022 [1]. While localized PCa is often curable, advanced PCa poses a significant challenge due to its high risk of biochemical recurrence (BCR), a critical precursor to clinical progression, local recurrence, and distant metastasis [2,3]. Current clinical models, reliant on serum prostate-specific antigen (PSA) levels, Gleason scores, and TNM staging, suffer from limited predictive accuracy, failing to capture the complex tumor biology of advanced PCa [2,3,4,5]. This unmet need for precise, individualized prognostic tools underscores the urgency of developing advanced methods to enhance cancer progression prediction and optimize patient outcomes.

Multiparametric MRI (mpMRI) has become a cornerstone for identifying PCa patients at risk of recurrence, leveraging its ability to visualize tumor characteristics non-invasively [6,7,8,9,10,11,12,13,14]. However, its application in advanced PCa is underexplored, and current MRI-based workflows face significant limitations: (1) qualitative assessments are prone to interobserver variability, compromising reproducibility [7,8], and (2) manual measurements of tumor volume, apparent diffusion coefficient (ADC) values, and lesion boundaries are time-consuming and lack standardization [9]. These challenges hinder the integration of imaging biomarkers into robust predictive models, limiting their clinical utility in guiding personalized treatment for advanced PCa.

Recent advancements have extended the application of deep learning beyond radiological imaging to include high-precision pathological assessment. For instance, Devnath et al. [15] recently developed an integrated machine learning network to accurately recognize epithelial cells within prostatic glands, demonstrating the potential of artificial intelligence (AI) to mitigate inter-observer variability in histopathological grading. These multi-scale AI-driven approaches collectively aim to standardize clinical workflows and provide more objective prognostic insights.

Deep learning-based radiomics has shown promise in predicting 24-month progression in advanced PCa using pretreatment mpMRI-derived features, surpassing traditional radiomics and clinical models in accuracy [16,17]. By extracting quantitative imaging features, radiomics captures subtle tumor characteristics missed by conventional methods. However, two critical gaps remain. First, the reliability of radiomic features depends heavily on region-of-interest (ROI) annotation, yet the impact of manual (ROIref) versus AI-driven (ROIai) tumor segmentation on feature stability and predictive performance remains unquantified [9]. Second, while radiomics excels at binary progression prediction, its potential to estimate time-to-progression—a key factor in determining optimal timing for salvage therapies—has not been explored. Addressing these gaps is essential to developing scalable, observer-independent tools for precision oncology.

To bridge these gaps, we developed and validated a deep learning-based radiomics model trained on manually annotated ROIs (ROIref) and tested on AI-segmented ROIs (ROIai) to predict progression in advanced PCa. We also assessed the prognostic value of radiomics-derived risk scores for estimating time-to-progression through survival analysis. By demonstrating the equivalence of the ROIai-based approach to the ROIref-based approach and extending radiomics to temporal risk stratification, this study establishes a robust, automated framework to enhance progression prediction, reduce reliance on labor-intensive manual annotations, and guide risk-adapted therapeutic strategies for improved patient outcomes.

## 2. Materials and Methods

### 2.1. Data Enrollment

This retrospective study was approved by the local Institutional Review Board (IRB number: 2021-342) with a waiver for written informed consent. Patients were enrolled from October 2017 to March 2024. All patients were diagnosed with PCa through ultrasound-guided systematic prostate biopsy. Inclusion criteria required pretreatment MRI scans in the Picture Archiving and Communication System (PACS), initial treatment with radiation therapy (RT), hormone therapy (HT), chemotherapy or a combination, regular follow-up (every 3 months in the first year and every 6 months thereafter), complete clinical records, and a minimum follow-up of 24 months with documented progression or non-progression. Based on the initial 2232 cases in our previous study [16,17], 182 cases were finally included in current study and stratified into Cohort 1 (follow-up at 24 months; *n* = 139) and Cohort 2 (follow-up > 24 months; *n* = 43). Treatment modalities: 94 cases received RT alone or RT with HT; 74, HT alone; 10, HT combined with chemotherapy; and 4, multiple-line sequential therapy.

### 2.2. Image Scanning Protocols

All MR data were acquired using 11 scanners from four different vendors. No statistically significant differences were observed between Cohort 1 and Cohort 2 in scanner manufacturers, field strengths, or most acquisition parameters (*p* > 0.05). Detailed scanning parameters are provided in Table 1.

### 2.3. Clinical Information and Reference Standard for Cancer Progression

Time to cancer progression was calculated as the months elapsed from treatment to the occurrence of progression or the last follow-up. At the end of the follow-up period, the treatment method was recorded as either single (HT only or RT only) or multiple (combination of two or more methods).

The primary endpoint of this study was progression-free survival, defined as the time from treatment to the first documented progression event. Progression was operationally defined as a composite endpoint encompassing biochemical, radiologic, or clinical progression, whichever occurred first. Biochemical progression was defined according to established criteria based on the initial treatment modality. For patients receiving HT, biochemical progression was defined using the Phoenix definition (PSA nadir + 2 ng/mL) [2]. For patients treated with androgen deprivation therapy (ADT), biochemical progression was defined according to the American Urological Association (AUA) criteria. Among patients receiving HT alone, progression was further characterized by the development of castration-resistant prostate cancer (CRPC). CRPC was defined as castrate serum testosterone levels (<1.7 nmol/L) in combination with either (1) three consecutive rises in PSA (with intervals of ≥1 week), resulting in a >50% increase over the PSA nadir and a PSA level exceeding 2 ng/mL [18], or (2) radiologic evidence of new metastatic lesions [16,17,19,20]. Radiologic progression was defined as the appearance of new metastatic lesions on follow-up imaging. Clinical progression was defined as the development of disease-related symptoms or complications attributable to disease progression, as documented by the treating physician.

To ensure comparability across treatment subgroups, all progression events were harmonized into a single composite endpoint, and time to progression was calculated uniformly regardless of the type of progression event.

### 2.4. ROI Annotation

Based on findings from prior research [16], we extracted imaging features from ROIs corresponding to PCa areas visible on mpMRI images. Two distinct ROI annotation methods were employed. Method 1 (ROIref): Two genitourinary radiologists (H.W., 15 years of experience; X.W., 30 years of experience) annotated ROIs by synthesizing information from diffusion-weighted imaging (DWI), ADC maps, T2-weighted imaging (T2WI), and dynamic contrast-enhanced (DCE) sequences (when available). The lesion with the highest Prostate Imaging Reporting and Data System (PI-RADS) score [21] was selected. Discrepancies between radiologists were resolved through consensus, establishing ROIref as the reference standard. Method 2 (ROIai): A pretrained deep learning model for PCa segmentation [22] was used to automatically identify ROIai. This model, based on a cascade 3D U-Net architecture, was trained on a large dataset (*n* = 1428 patients from 7 MRI scanners across 4 vendors at both 1.5T and 3.0T field strengths) and validated for detecting clinically significant PCa on mpMRI, achieving a Dice similarity coefficient of 0.69 ± 0.28 and patient-level sensitivity of 90.0% in patients with PSA levels of 4–10 ng/mL. For each patient in the current study, the model automatically segmented all suspected lesions on ADC maps, and the largest predicted lesion within the prostate was selected as ROIai, consistent with our selection of the highest PI-RADS lesion in ROIref. Notably, the ROIai were generated in a fully automated manner without any manual correction to rigorously assess the model’s tolerance to segmentation variability. No complete segmentation failures (i.e., failure to detect a lesion) occurred in the final 182-patient cohort.

Imaging features were extracted from ROIs corresponding to PCa areas visible on mpMRI images. For patients with multifocal disease, only the index lesion was analyzed. For ROIref, the lesion with the highest PI-RADS score was selected by the consensus of two radiologists after reviewing all available sequences. In cases where multiple lesions shared the same highest PI-RADS score, the one with the largest volume was selected. For ROIai, the pretrained model automatically identified the largest predicted lesion volume as the target ROI.

The progression prediction radiomics model was trained using ROIref-derived features. Once established, the radiomics model was applied to ROIai to predict progression probabilities. Predictive outcomes from both ROIref-based approach and ROIai-based approach were systematically compared.

To compare measured data of ROIref and ROIai, volumetric measurements (volume), dimensional parameters (RL, AP, and SI diameters), and ADC values were quantified and compared for each case. Spatial overlap between ROIref and ROIai was assessed using the Dice similarity coefficient (DSC), volume similarity (VS), and average Hausdorff distance (HD).

### 2.5. Progression Prediction Model Development

**Figure 1:**
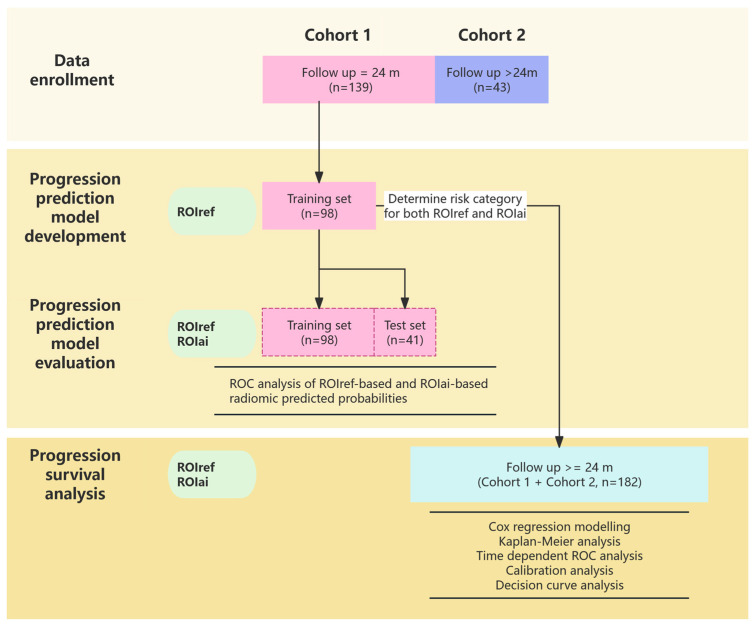
Flowchart illustrating the systematic development and evaluation of a prostate cancer progression prediction model using two radiomics features (ROIref and ROIai). ROI, region of interest; ROIref, manually labeled ROI; ROIai, AI derived ROI; ROC, receiver operating characteristic.

The data in Cohort 1 (*n* = 139) were randomly divided into a training set (*n* = 98) and an independent test set (*n* = 41) at a ratio of 7:3. We developed a deep learning-based radiomics model from pretreatment MRI ADC maps with ROIref annotations to predict progression within 24 months in advanced PCa patients using the training set. The data split method is illustrated in Figure 1.

To mitigate scanner variability, ADC maps underwent intensity normalization during preprocessing. Three distinct image sets were analyzed (Supplementary Figure S1): (1) Original Images (unprocessed ADC maps), (2) LoG Images (processed with a Laplacian of Gaussian filter to enhance edge details), and (3) Wavelet Images (generated via 3D wavelet decomposition using the PyWavelet package across the *x*, *y*, and *z* axes).

ROIs were resampled to a uniform spatial resolution to standardize input dimensions. A pre-trained MedicalNet architecture [23], initialized with weights pretrained on large-scale medical imaging datasets, was employed to extract deep features. The MedicalNet architecture is a 3D extension of the ResNet family specifically designed for medical imaging applications. It was pre-trained on the 3DSeg-8 dataset (1638 3D medical volumes from 8 segmentation tasks covering multiple organs and imaging modalities including CT and MRI). Using features learned from diverse medical imaging data, MedicalNet provides better initialization for medical imaging tasks compared to natural image pre-trained models or training from scratch. Transfer learning from MedicalNet has been shown to accelerate training convergence by 2–10 times and improve accuracy by 3–20% across various 3D medical imaging applications. The model’s convolutional layers processed ROIs to generate channel-wise feature maps, which underwent global max pooling to reduce dimensionality, yielding 2048 one-dimensional features per ROI.

Subsequent feature engineering included z-score normalization followed by principal component analysis (PCA), retaining 95% of the cumulative variance to reduce dimensionality and minimize redundancy. Given the high dimensionality of the deep learning-derived features (*n* = 2048), PCA was performed exclusively on the standardized feature matrix of the training cohort, and the resulting transformation was subsequently applied to the test cohort without refitting. The number of retained principal components was determined based on the cumulative explained variance.

From the PCA-reduced feature space, the eight most discriminative features were selected using a combination of statistical significance testing (comparing progression versus non-progression groups) and correlation analysis, with highly correlated redundant features removed (|r| > 0.9). These final eight deep learning-derived features were then used as inputs to a logistic regression classifier optimized with L2 regularization. Unlike traditional radiomics approaches that rely on handcrafted features (e.g., texture, shape, and intensity statistics), the proposed deep learning-based radiomics framework automatically learns hierarchical feature representations directly from ADC maps through a convolutional neural network.

Model performance was evaluated in the training set using stratified 5-fold cross-validation, ensuring each fold preserved the progression class distribution.

### 2.6. Progression Prediction Model Evaluation

The progression prediction radiomics model was trained using deep learning-derived features extracted exclusively from ROIref in the training subset and was subsequently evaluated on both the training and independent test subsets using features derived from ROIref and ROIai. Once established, the same trained radiomics model was applied to features extracted from both ROIref and ROIai in the test subset to predict progression probabilities. Predictive outcomes obtained using the two ROI annotation methods were then systematically compared. Importantly, the feature extraction approach (deep learning-based) and the trained model were identical for both ROI types; only the source of tumor delineation differed (Figure 1 and Supplementary Figure S2).

The discrimination ability of the ROIref- and ROIai-derived probability scores was quantified using receiver operating characteristic (ROC) analysis, with the area under the curve (AUC) calculated for both ROI types. Statistical differences between the AUC values were assessed using the DeLong test.

### 2.7. Survival Analysis

Survival analysis was conducted to evaluate the time to progression in the entire enrolled data using both ROIref and ROIai. Covariates were selected based on prior evidence linking them to adverse prognosis and included age, baseline PSA, biopsy Gleason grade, clinical TNM stage, and mpMRI findings [4,16,17,24]. Additionally, the radiomics model’s predicted risk probabilities were evaluated as covariates.

For risk stratification, ROIref-based prediction probabilities from the training subset (*n* = 98, 70% of Cohort 1) were used to determine an optimal cutoff for high- versus low-risk classification via the Youden index. This cutoff was subsequently applied to categorize all 182 patients for ROIref-based and ROIai-based approaches. Survival differences between risk groups were visualized using Kaplan–Meier curves.

Univariable Cox regression analysis was conducted to assess associations between covariates (clinical variables and radiomics-derived probabilities from ROIref/ROIai) and progression risk. Variables showing a trend toward significance (*p* < 0.10) in univariable analysis were retained for multivariable modeling. Two multivariable Cox proportional hazards models were then constructed: Model 1 combined ROIref-derived probabilities with significant clinical variables, while Model 2 used ROIai-derived probabilities with the same clinical covariates. Both models reported hazard ratios (HRs) with 95% confidence intervals (CIs).

Prognostic performance was evaluated using three metrics: discrimination, calibration, and clinical utility. Discrimination was quantified via the concordance index (C-index), which measures the agreement between predicted probabilities and observed progression events. Calibration was assessed by plotting observed versus predicted progression incidences at 12, 24, 36, and 48 months, with Brier scores calculated using bootstrap cross-validation (1000 iterations) to evaluate prediction accuracy. Finally, clinical utility was appraised using decision curve analysis (DCA) [25], which quantified the net benefit of ROIref-based method and ROIai-based method across risk thresholds spanning 12 to 48 months, thereby informing their applicability in clinical decision-making.

To assess the robustness of the ROIai-predicted probabilities across different clinical subgroups, subgroup analyses based on International Society of Urological Pathology (ISUP) grade, cT stage, cN stage, cM stage, and treatment category were conducted with interaction testing.

### 2.8. Statistical Analysis

All statistical analyses were performed using R software (version 4.3.1; http://www.r-project.org). Continuous variables are reported as medians with interquartile ranges (IQR), and categorical variables are summarized as frequencies with percentages [*n* (%)]. Differences in continuous variables between groups were assessed using the Mann–Whitney U test, while categorical variables were compared using the chi-square test or Fisher’s exact test, as appropriate. A two-tailed *p* value < 0.05 was considered statistically significant.

## 3. Results

### 3.1. Patient Demographics and PCa Characteristics

Among 182 enrolled patients, the non-progression group showed significantly older age (71.6 ± 8.0 years vs. 70.7 ± 8.3 years, *p* < 0.001) and lower serum PSA levels (median 36.5 [IQR 14.2, 99.7] ng/dL vs. 82.5 [20.4, 290.0] ng/dL, *p* = 0.016) compared to the progression group. Imaging revealed smaller maximum lesion diameter (3.5 [2.5, 4.8] cm vs. 4.8 [3.5, 6.0] cm, *p* < 0.001) and reduced lesion volume (10.0 [3.4, 22.0] cm^3^ vs. 26.4 [8.1, 51.8] cm^3^, *p* < 0.001) in non-progression group. The proportion of ISUP grade 5 on biopsy was significantly lower in non-progression group (44.7% [55/123] vs. 67.8% [40/59], *p* = 0.045). Clinically, non-progression group demonstrated higher rates of N0 staging (68.3% [84/123] vs. 37.3% [22/59]) and M0 staging (66.7% [82/123] vs. 30.5% [18/59]), whereas progression group predominated in N1 (62.7% [37/59] vs. 31.7% [39/123]) and M1 (69.5% [41/59] vs. 33.3% [41/123]) classifications (both *p* < 0.001). Multimodal therapy was more frequently administered in non-progression group (74.8% [92/123] vs. 23.7% [14/59], *p* < 0.001). No significant differences were observed in PI-RADS scores, lesion number, ADC values, or cT staging (all *p* > 0.05), as detailed in Table 2.

### 3.2. Comparison of ROIref and ROIai

The comparative analysis between ROIref and ROIai measurements demonstrated strong agreement across quantitative parameters in the overall cohort. No statistically significant differences were observed in volumetric measurements (15.2 vs. 14.1 cm^3^, *p* = 0.935), ADC values (0.774 vs. 0.781 × 10^3^ mm^2^/s, *p* = 0.927), or dimensional assessments including RL (3.8 vs. 3.78 cm, *p* = 0.788), AP (3.5 vs. 3.5 cm, *p* = 0.923), and SI diameters (4.0 vs. 4.0 cm, *p* = 0.543). Segmentation accuracy metrics revealed excellent spatial correspondence, with median DSC of 0.901, vs. of 0.953, and HD of 0.184 mm. Subgroup analyses demonstrated comparable results in both Cohort 1 and Cohort 2. Detailed results are presented in Table 3.

### 3.3. Progression Classification Efficacy

**Figure 2:**
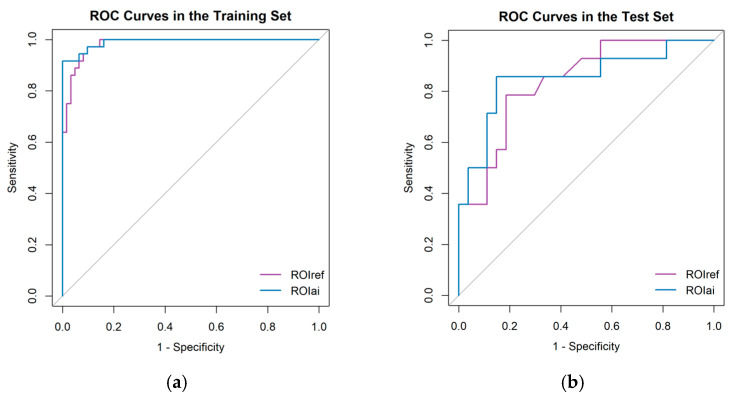
Comparison of the AUC values of the ROC curves for the radiomics model in predicting progression using ROIref and ROIai methods in the training cohort (**a**) and test cohort (**b**). AUC, area under the curve.

Model performance was assessed using AUC (Figure 2). In the training set, ROIref-based method achieved an AUC of 0.983 (95% CI: 0.964–1.000), while ROIai-based method yielded an AUC of 0.991 (95% CI: 0.979–1.000), with no significant difference (DeLong test, *p* = 0.338). Similarly, in the test set, ROIref-based method and ROIai-based method showed comparable discrimination, with AUCs of 0.840 (95% CI: 0.717–0.963) and 0.852 (95% CI: 0.712–0.991), respectively. The DeLong test confirmed no statistically significant difference between ROIai-based and ROIref-based approaches in the test set (*p* = 0.870).

Additional performance metrics including accuracy, sensitivity, specificity, positive predictive value, and negative predictive value are detailed in Table 4.

### 3.4. Survival Analysis

**Figure 3:**
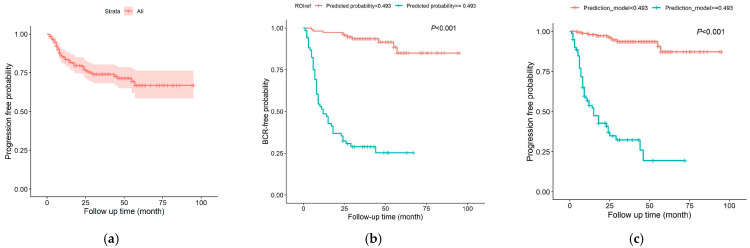
Kaplan–Meier curve for progression-free probability in the overall cohort (**a**) and stratified by radiomics-derived risk groups using ROIref (**b**) and ROIai (**c**).

The median follow-up duration for all patients was 34.0 months (IQR 24.0–39.0). Cancer progression occurred in 59 patients, with 38 cases (64.4%) emerging within 12 months of initial treatment. Subsequent intervals showed declining incidence: 12 patients (20.3%) experienced progression between 12–24 months, 5 (8.5%) between 24–36 months, 2 (3.4%) between 36–48 months, and 2 (3.4%) beyond 48 months. The estimated non-progression rates at 12, 24, 36, and 48 months were 79.1%, 72.5%, 69.4%, and 67.0%, respectively (Figure 3a).

Univariate Cox regression analysis identified several significant prognostic factors for progression: nodal involvement (cN1 vs. cN0: HR = 2.97, 95% CI = 1.75–5.05; *p* < 0.001), distant metastasis (cM1 vs. cM0: HR = 3.56, 95% CI = 2.04–6.21; *p* < 0.001), single treatment modality (single vs. combination therapy: HR = 7.96, 95% CI = 4.27–14.90; *p* < 0.001), and elevated radiomics-based prediction probabilities (ROIref: HR = 17,076, 95% CI = 1751–166,520; ROIai: HR = 38,022, 95% CI = 3447–419,455; both *p* < 0.001). Notably, ISUP grade 4–5 (4–5 vs. 1–3: HR = 2.09, 95% CI = 0.99–4.41; *p* = 0.035) and PSA levels (HR = 1.00 per unit increase, 95% CI = 1.00–1.001; *p* = 0.033) demonstrated borderline significance, though their confidence intervals marginally overlapped the null value. Age, PI-RADS classification, and advanced clinical T-stage were not significantly associated with outcomes (Table 5).

In multivariate analyses, both ROIref- and ROIai-predicted progression probabilities retained independent prognostic value (both *p* < 0.001) after adjustment for clinical covariates (cN1, cM1, treatment modality, ISUP grade, and PSA). Proportional hazards (PH) assumptions were satisfied for ROIref (*p* = 0.114) and ROIai (*p* = 0.436) via Schoenfeld residual testing. Model performance evaluation revealed strong discriminative ability, with concordance indices (C-indices) of 0.842 (95% CI = 0.799–0.885) for the ROIai-precited progression probabilities and 0.833 (95% CI = 0.794–0.872) for the ROIref-predicted progression probabilities.

**Figure 4:**
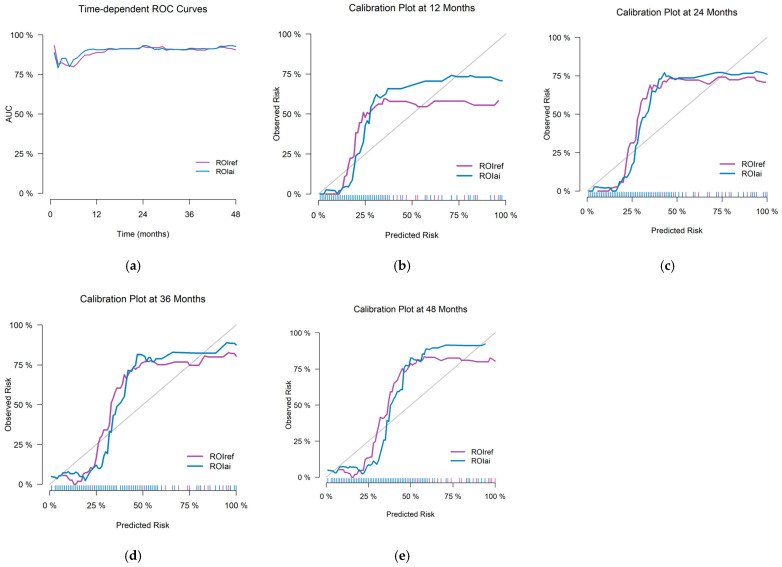
Time-dependent ROC curves for ROIref- and ROIai-predicted probabilities over 48 months (**a**) and calibration plots for ROIref- and ROIai-predicted probabilities at 12 (**b**), 24 (**c**), 36 (**d**), and 48 months (**e**).

Time-dependent ROC curves (Figure 4a) and calibration plots at 12, 24, 36, and 48 months (Figure 4b–e) demonstrated comparable performance between the ROIref-based and ROIai-based approaches. Both achieved similar AUC values and Brier scores across all timepoints (Table 6), with no statistically significant differences observed (*p* > 0.05 for all comparisons).

Kaplan–Meier survival analysis was conducted to assess the binary classification efficacy of ROIref- and ROIai-based predicted probabilities in stratifying progression risk. Using the Youden index-derived cutoff (probability ≥ 0.493 for high-risk classification), both methods demonstrated significant separation between high-risk (predicted probability ≥ 0.493) and low-risk (predicted probability < 0.493) groups (log-rank *p* < 0.001 for both; Figure 3b,c).

**Figure 5:**
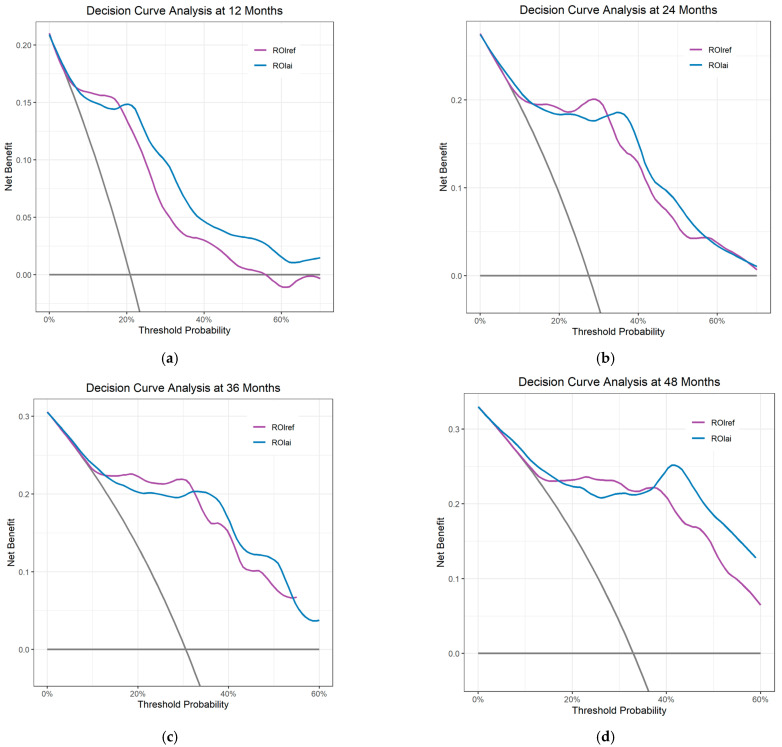
Decision curve analysis evaluating clinical utility of ROIref- and ROIai-radiomics-predicted probability for progression risk stratification at 12 (**a**), 24 (**b**), 36 (**c**), and 48 months (**d**).

DCA was performed to compare the clinical utility of ROIref-predicted and ROIai-predicted progression probabilities in guiding progression risk stratification. As illustrated in Figure 5, net benefit curves for both approaches were evaluated at 12, 24, 36, and 48 months across a clinically relevant range of threshold probabilities (0.1–0.6). At all time points, the net benefit of ROIref-bashed method and ROIai-based method exceeded the “treat all” and “treat none” reference strategies, confirming their potential to improve clinical decision-making. The curves for ROIref-based and ROIai-based approaches exhibited overlapping trajectories and crossovers, with neither method demonstrating consistent superiority.

**Figure 6:**
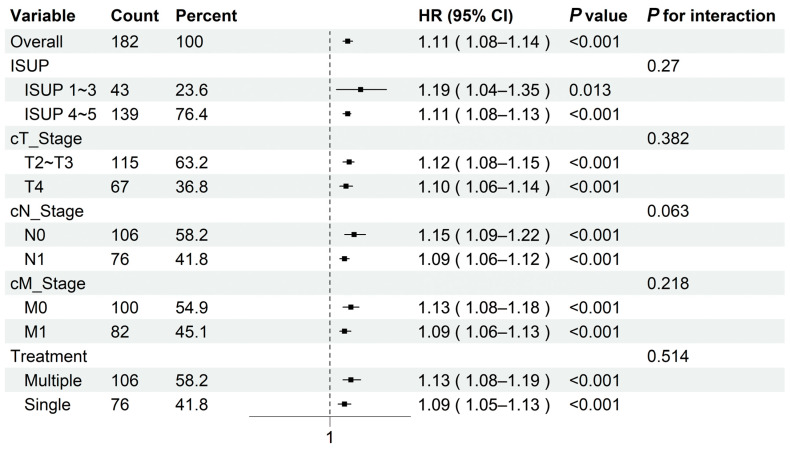
Forest plot of stratified subgroup and interaction analyses assessing the robustness of the ROIai-based progression prediction across different clinical subgroups.

In sensitivity analyses stratified by clinical characteristics, the ROIai-based radiomics prediction probability showed consistent association with progression risk across subgroups (Figure 6). Effect estimates remained consistent across ISUP grade, tumor status, nodal status, metastatic status and treatment modality, with no significant interactions detected (all *p* for interaction > 0.05). These findings indicate that the radiomics model provides stable prognostic value irrespective of key clinical characteristics.

## 4. Discussion

Our study demonstrated that AI-derived tumor segmentation achieves diagnostic and prognostic parity with manual expert annotations in advanced PCa. As a scalable, observer-independent tool for precision risk assessment, AI-driven radiomics could reduce reliance on labor-intensive manual workflows without compromising diagnostic or prognostic accuracy.

Our study addresses critical gaps in the progression prediction literature through two key distinctions. First, unlike prior investigations focusing localized PCa [11,12,13,14,26], our study specifically targeted advanced PCa patients undergoing non-surgical therapies. This distinction is biologically significant, as recurrence mechanisms in advanced PCa—shaped by treatment-induced microenvironmental changes (e.g., hypoxia, androgen receptor alterations)—differ fundamentally from postoperative recurrence driven by residual tumor burden. Second, while existing models rely on labor-intensive manual evaluations of preoperative MRI that are prone to interobserver variability and inefficiency, our AI-driven radiomics framework automates feature extraction, reducing subjectivity (Dice = 0.901 for AI vs. manual ROIs) and analysis time.

Building on our team’s earlier work [16,17], this study introduces three methodological advancements. (1) Enhanced Validation: Validation in an expanded cohort (*n* = 182 vs. prior *n* = 131) with extended follow-up (median 34 months vs. 24 months). (2) ROI Robustness Analysis: First direct comparison of manual and AI-generated segmentation impacts, demonstrating equivalent prognostic value and resolving prior concerns about annotation dependency. These findings support the feasibility of replacing manual tumor delineation with automated AI-based segmentation in radiomics-driven progression prediction, without compromising predictive accuracy. (3) Temporal Prognostication: Beyond binary progression prediction, we established radiomics as a time-to-event predictor, with stable discrimination (48-month AUC > 0.75) and calibration (Brier score < 0.15), enabling risk-adapted surveillance intervals—a capability absent in earlier models. These innovations collectively advance radiomics toward clinically actionable, observer-agnostic tools for precision oncology.

The equivalent prognostic performance of AI-based and manual segmentation represents a significant step toward observer-agnostic precision oncology. By resolving the dependency on manual annotation—a primary source of variability and a major hurdle in clinical workflows—our findings demonstrate that deep learning radiomics can be transitioned from a research tool into a scalable, automated clinical decision support system. This ensures that the high predictive accuracy is not only achievable in a controlled study environment but also reproducible in real-world clinical practice where time and expert resources are limited.

Radiomics studies should adhere to technical guidelines to enhance research quality [27,28,29,30,31]. These guidelines emphasize the formal evaluation of fully automated segmentation [9,27]. In this study, our segmentation model has demonstrated high performance, validated across multiple studies [22,32]. Beyond prior validation, we directly compared AI-segmented ROIs with expert-manually annotated ROIs in this study. The results showed good consistency between the two, likely due to the well-defined, larger-volume lesions in our cohort. Previous research has shown that AI can more accurately detect lesions with lower ADC values and larger volumes [33]. Given the relatively simple task for AI in this study, these results are promising for future applications. Automated AI segmentation not only eliminates this coordination challenge but also significantly reduce waiting time and improve clinical efficiency.

In this study, logistic regression was utilized as the final classifier to integrate the selected deep learning features. This choice was motivated by its robustness and resistance to overfitting, particularly in radiomics studies where the feature-to-sample ratio must be carefully managed. Unlike ‘black-box’ machine learning algorithms, logistic regression offers high clinical interpretability, providing a transparent relationship between imaging phenotypes and the probability of cancer progression [34]. Furthermore, its performance was found to be equivalent to more complex classifiers in our pilot study, consistent with previous reports suggesting that model simplicity often enhances reproducibility in medical imaging AI [35]. In the model-selection phase of this study, various machine learning classifiers, including Support Vector Machine and Random Forest, were evaluated. Since these complex algorithms did not significantly outperform the linear model, logistic regression was finalized as the classifier to ensure the highest degree of model stability and interpretability, consistent with the preference for simpler models in clinical prognostic research [36].

The survival analysis further supports the clinical relevance of the proposed radiomics-based progression prediction model. Specifically, the HRs derived from the Cox regression analyses indicate that increasing radiomics-predicted probabilities are associated with a substantially higher risk of disease progression over time, providing an interpretable measure of relative risk rather than a simple binary classification. Based on the predefined risk stratification threshold, patients could be separated into high-risk and low-risk groups with clearly distinct progression-free survival curves, suggesting potential utility for individualized surveillance strategies and risk-adapted clinical management.

Importantly, the model demonstrated stable time-dependent performance throughout follow-up, with sustained discrimination (time-dependent AUC values exceeding 0.75 up to 48 months) and good calibration, indicating consistent prognostic value across clinically relevant time horizons. This temporal robustness suggests that the radiomics signature captures biologically meaningful imaging features associated with disease progression, rather than reflecting short-term or time-specific effects. Together, these findings highlight the potential of radiomics-based survival modeling to support longitudinal risk assessment in advanced PCa.

This study has several limitations. First, its retrospective, single-center design and modest sample size risk selection bias and may limit generalizability, particularly in underrepresented subgroups. While the model showed high performance in our independent test set, variations in MRI scanners and imaging protocols across different centers could impact the stability of radiomic features. Future studies involving multi-center cohorts are warranted to evaluate the robustness and clinical utility of our model in more diverse clinical settings. Advanced harmonization methods, such as ComBat, could also be applied to improve feature stability in future multi-center studies. Second, heterogeneous treatment protocols were not rigorously controlled, potentially confounding progression risk estimates. Although treatment-stratified subgroup and interaction analyses demonstrated consistent prognostic effects of the radiomics-based model, residual confounding related to treatment heterogeneity and unmeasured treatment intensity (e.g., duration of ADT) cannot be completely excluded in this retrospective cohort. Future prospective studies with standardized treatment protocols and detailed treatment exposure data are therefore necessary to further optimize and validate radiomics-based progression prediction models. Third, while AI-derived radiomics demonstrated parity with manual annotations, the model’s generalizability may be constrained by scanner variability and vendor-specific ADC quantification biases. Additionally, the lack of integration with multimodal biomarkers (e.g., genomics, PSMA-PET) and real-world validation of clinical workflow integration represent critical gaps. Future multicenter studies with standardized imaging protocols, treatment-stratified analyses, and prospective validation are needed to translate these findings into robust, clinically actionable tools.

## 5. Conclusions

In conclusion, this study demonstrates that AI-derived radiomics features achieve diagnostic and prognostic performance equivalent to expert manual annotations in predicting advanced PCa progression. Despite limitations in sample size and retrospective design, these findings underscore the transformative potential of AI-enhanced radiomics for precision risk stratification.

## Figures and Tables

**Table 1:** Image acquisition protocols.

	Overall (*n* = 182)	Cohort 1 (*n* = 139)	Cohort 2 (*n* = 43)	*p* Value
Manufacture				0.122
GE ^a^	100 (54.9%)	70 (50.4%)	30 (69.8%)	
PHILIPS ^b^	39 (21.4%)	32 (33.0%)	7 (16.3%)	
SIEMENS ^c^	34 (18.7%)	29 (20.9%)	5 (11.6%)	
UIH ^d^	9 (4.9%)	8 (5.8%)	1 (2.3%)	
Model Name				0.293
DISCOVERY MR750	93 (51.1%)	65 (46.8%)	28 (65.1%)	
SIGNA EXCITE	7 (3.8%)	5 (3.6%)	2 (4.7%)	
ACHIEVA	12 (6.6%)	9 (6.5%)	3 (7.0%)	
INGENIA	16 (8.8%)	15 (10.8%)	1 (2.3%)	
MULTIVA	11 (6.0%)	8 (5.8%)	3 (7.0%)	
AERA	34 (18.7%)	29 (20.9%)	5 (11.6%)	
uMR 790	9 (4.9%)	8 (5.8%)	1 (2.3%)	
Magnetic Field Strength				0.226
1.5 T	49 (26.9%)	41 (29.5%)	8 (18.6%)	
3.0 T	133 (73.1%)	98 (70.5%)	35 (81.4%)	
Reconstruction Diameter (mm)				0.159
Median [Q1, Q3]	240 [200, 240]	240 [200, 240]	240 [210, 240]	
Slice Thickness (mm)				0.016
Median [Q1, Q3]	4.0 [4.0, 4.0]	4.0 [4.0, 4.0]	4.0 [4.0, 4.5]	
Repetition Time (ms)				0.058
Median [Q1, Q3]	3000 [2640, 5010]	3030 [2640, 5010]	2670 [2630, 3500]	
Echo Time (ms)				0.152
Median [Q1, Q3]	61.0 [55.5, 61.8]	60.9 [54.2, 61.8]	61.2 [60.5, 63.0]	
Pixel Bandwidth (MHz)				0.713
Median [Q1, Q3]	1950 [1790, 1950]	1950 [1770, 1950]	1950 [1950, 1950]	
Flip Angle (°)				0.176
Median [Q1, Q3]	90 [90, 90]	90 [90, 90]	90 [90, 90]	
B Value (s/mm^2^)				0.481
800	11 (6.0%)	8 (4.4%)	3 (7.0%)	
1000	2 (1.1%)	1 (0.7%)	1 (2.3%)	
1200	4 (2.2%)	3 (2.2%)	1 (2.3%)	
1400	165 (90.7%)	127 (91.4%)	38 (88.4%)	

^a^ Discovery HD 750, Ge Healthcare, Milwaukee, WI, USA & Signa Excite, GE Healthcare, Milwaukee, WI, USA. ^b^ Achieva TX, Philips Healthcare, Best, The Netherlands & Ingenia Philips Healthcare, Best, The Netherlands & Multiva, Philips Healthcare, Best, The Netherlands. ^c^ Aera, Siemens Healthcare, Erlangen, Germany. ^d^ uMR 790, United Imaging Healthcare, Shanghai, China.

**Table 2:** Clinical characteristics.

	Overall (*n* = 182)			Cohort 1 (*n* = 139)			Cohort 2 (*n* = 43)		
	Non-Progression	Progression	*p* Value	Non-Progression	Progression	*p* Value	Non-Progression	Progression	*p* Value
	(*n* = 123)	(*n* = 59)		(*n* = 89)	(*n* = 50)		(*n* = 34)	(*n* = 9)	
Age (year)			<0.001			<0.001			<0.001
Mean (SD)	71.6 (8.0)	70.7 (8.3)		71.2 (8.5)	71.2 (8.1)		72.7 (6.7)	67.7 (9.2)	
PSA (ng/dL)			0.016			0.071			0.105
Median [Q1, Q3]	36.5 [14.2, 99.7]	82.5 [20.4, 290.0]		42.6 [14.3, 105.0]	62.8 [19.3, 289.0]		27.7 [12.3, 59.7]	132.0 [34.4, 262.0]	
PI-RADS			>0.999			>0.999			>0.999
PI-RADS 4	3 (2.4%)	1 (1.7%)		2 (2.2%)	1 (2.0%)		1 (2.9%)	0 (0%)	
PI-RADS 5	120 (97.6%)	58 (98.3%)		87 (97.8%)	49 (98.0%)		33 (97.1%)	9 (100%)	
Lesions Number			0.656			0.594			>0.999
1	116 (94.3%)	57 (96.6%)		82 (92.1%)	48 (96.0%)		34 (100%)	9 (100%)	
2	4 (3.3%)	1 (1.7%)		4 (4.5%)	1 (2.0%)		0 (0%)	0 (0%)	
3	2 (1.6%)	0 (0%)		2 (2.2%)	0 (0%)		0 (0%)	0 (0%)	
4	1 (0.8%)	1 (1.7%)		1 (1.1%)	1 (2.0%)		0 (0%)	0 (0%)	
Lesions Diameter (cm)			<0.001			0.001			0.034
Median [Q1, Q3]	3.5 [2.5, 4.8]	4.8 [3.5, 6.0]		3.4 [2.6, 4.8]	4.7 [3.5, 5.9]		3.6 [2.4, 4.3]	5.3 [3.9, 6.4]	
Lesion Volume (cm^3^)			<0.001			0.001			0.026
Median [Q1, Q3]	10.0 [3.4, 22.0]	26.4 [8.1, 51.8]		10.8 [3.4, 22.1]	26.1 [7.9, 44.8]		9.7 [2.6, 19.5]	44.5 [13.9, 66.8]	
ADC Value (×10^3^ mm^2^/s)			0.718			0.444			0.547
Median [Q1, Q3]	0.773 [0.727, 0.879]	0.778 [0.729, 0.851]		0.774 [0.730, 0.880]	0.774 [0.728, 0.853]		0.764 [0.725, 0.840]	0.813 [0.733, 0.821]	
ISUP			0.045			0.063			0.485
1	3 (2.4%)	1 (1.7%)		1 (1.1%)	1 (2.0%)		2 (5.9%)	0 (0%)	
2	11 (8.9%)	1 (1.7%)		7 (7.9%)	0 (0%)		4 (11.8%)	1 (11.1%)	
3	21 (17.1%)	6 (10.2%)		16 (18.0%)	6 (12.0%)		5 (14.7%)	0 (0%)	
4	33 (26.8%)	11 (18.6%)		26 (29.2%)	10 (20.0%)		7 (20.6%)	1 (11.1%)	
5	55 (44.7%)	40 (67.8%)		39 (43.8%)	33 (66.0%)		16 (47.1%)	7 (77.8%)	
cT Stage			0.220			0.251			0.815
T2	7 (5.7%)	3 (5.1%)		5 (5.6%)	2 (4.0%)		2 (5.9%)	1 (11.1%)	
T3	76 (61.8%)	29 (49.2%)		54 (60.7%)	24 (48.0%)		22 (64.7%)	5 (55.6%)	
T4	40 (32.5%)	27 (45.8%)		30 (33.7%)	24 (48.0%)		10 (29.4%)	3 (33.3%)	
cN Stage			<0.001			<0.001			>0.999
N0	84 (68.3%)	22 (37.3%)		64 (71.9%)	17 (34.0%)		20 (58.8%)	5 (55.6%)	
N1	39 (31.7%)	37 (62.7%)		25 (28.1%)	33 (66.0%)		14 (41.2%)	4 (44.4%)	
cM Stage			<0.001			0.001			0.023
M0	82 (66.7%)	18 (30.5%)		55 (61.8%)	15 (30.0%)		27 (79.4%)	3 (33.3%)	
M1	41 (33.3%)	41 (69.5%)		34 (38.2%)	35 (70.0%)		7 (20.6%)	6 (66.7%)	
Treatment			<0.001			<0.001			>0.999
Multiple	92 (74.8%)	14 (23.7%)		66 (74.2%)	7 (14.0%)		26 (76.5%)	7 (77.8%)	
Single	31 (25.2%)	45 (76.3%)		23 (25.8%)	43 (86.0%)		8 (23.5%)	2 (22.2%)	

**Table 3:** Comparison of manually labeled ROI (ROIref) and AI derived ROI (ROIai).

	Overall (*n* = 182)			Cohort 1 (*n* = 139)			Cohort 2 (*n* = 43)		
	ROIref	ROIai	*p* Value	ROIref	ROIai	*p* Value	ROIref	ROIai	*p* Value
Volume (cm^3^)									
Median [Q1, Q3]	15.2 [4.4, 30.7]	14.1 [5.1, 33.1]	0.935	15.6 [4.81, 31.0]	14.4 [5.12, 33.6]	0.955	12.9 [2.65, 28.0]	13.3 [2.63, 28.5]	0.806
ADC Value (×10^3^ mm^2^/s)									
Median [Q1, Q3]	0.774 [0.727, 0.860]	0.781 [0.717, 0.853]	0.927	0.774 [0.729, 0.862]	0.780 [0.724, 0.853]	0.823	0.765 [0.725, 0.836]	0.795 [0.707, 0.853]	0.894
RL Diameter (cm)									
Median [Q1, Q3]	3.8 [2.7, 4.8]	3.78 [2.6, 4.8]	0.788	3.8 [2.7, 4.8]	3.8 [2.7, 4.8]	0.678	3.7 [2.3, 4.6]	3.7 [2.4, 4.7]	0.928
AP Diameter (cm)									
Median [Q1, Q3]	3.5 [2.3, 4.7]	3.5 [2.3, 4.6]	0.923	3.6 [2.5, 4.6]	3.5 [2.4, 4.6]	0.883	3.1 [1.9, 4.8]	3.5 [1.9, 4.6]	0.959
SI Diameter (cm)									
Median [Q1, Q3]	4.0 [2.6, 5.2]	4.0 [2.8, 5.2]	0.543	4.0 [2.8, 5.2]	4.0 [2.8, 5.3]	0.725	3.6 [2.1, 4.8]	4.0 [2.8, 5.2]	0.525
DSC									
Median [Q1, Q3]	0.901 [0.853, 0.942]			0.902 [0.854, 0.942]			0.901 [0.856, 0.941]		
VS									
Median [Q1, Q3]	0.953 [0.908, 0.982]			0.956 [0.895, 0.984]			0.947 [0.918, 0.978]		
HD (mm)									
Median [Q1, Q3]	0.184 [0.095, 0.420]			0.186 [0.096, 0.441]			0.165 [0.084, 0.336]		

ROI, region of interest; VS, Volume similarity; DSC, Dice similarity coefficient; HD, Hausdorff distance.

**Table 4:** Evaluation metrics of the progression classification efficacy of the deep-radiomics model.

	AUC	ACC	SEN	SPE	PPV	NPV
Training set (*n* = 98)						
ROIref	0.983 (0.964, 1.000)	0.929 (0.927, 0.930)	0.972 (0.919, 1.000)	0.903 (0.830, 0.977)	0.854 (0.745, 0.962)	0.982 (0.948, 1.017)
ROIai	0.991 (0.979, 1.000)	0.969 (0.969, 0.970)	0.917 (0.826, 1.000)	1.000 (1.000, 1.000)	1.000 (1.000, 1.000)	0.954 (0.903, 1.005)
Test set (*n* = 41)						
ROIref	0.840 (0.717, 0.963)	0.805 (0.797, 0.812)	0.786 (0.571, 1.000)	0.815 (0.668, 0.961)	0.688 (0.460, 0.915)	0.880 (0.753, 1.007)
ROIai	0.852 (0.712, 0.991)	0.854 (0.848, 0.860)	0.857 (0.674, 1.000)	0.852 (0.718, 0.986)	0.750 (0.538, 0.962)	0.920 (0.814, 1.026)

AUC, area under the ROC curve; ACC, accuracy; SEN, sensitivity; SPE, specificity; PPV, positive predictive value; NPV, negative predictive value.

**Table 5:** Hazard ratios of Cox regression.

Variable	Univariate Cox Regression		Multivariate Cox Regression-ROIref		Multivariate Cox Regression-ROIai	
	HR (95% CI)	*p* Value	HR (95% CI)	*p* Value	HR (95% CI)	*p* Value
Age (year)	0.991 (0.960, 1.020)	0.561				
PSA (ng/dL)	1.000 (1.000, 1.001)	0.033				
PI-RADS						
PIRADS 4	ref	0.827				
PIRADS 5	1.240 (0.171, 8.930)					
ISUP						
ISUP 1~3	ref	0.035				
ISUP 4~5	2.090 (0.992, 4.410)					
cT Stage						
T2	ref	0.209				
T3	0.856 (0.261, 2.810)					
T4	1.380 (0.418, 4.550)					
cN Stage						
N0	ref	<0.001				
N1	2.970 (1.750, 5.050)					
cM Stage						
M0	ref	<0.001				
M1	3.560 (2.040, 6.210)					
Treatment						
Multiple	ref	<0.001				
Single	7.960 (4.270, 14.900)		4.030 (2.030, 7.990)	<0.001	4.470 (2.390, 8.380)	<0.001
ROIref-based predicted probability	17,100 (1750, 167,000)	<0.001	1390 (92.5, 20,875)	<0.001		
ROIai- based predicted probability	38,000 (3450, 419,000)	<0.001			20,618 (1232, 345,146)	<0.001

HR, Hazard ratios.

**Table 6:** AUC values and Breier Scores at different time points.

Time	AUC			Brier Score		
	ROIref	ROIai	*p* Value	ROIref	ROIai	*p* Value
12 month	0.888 (0.841, 0.934)	0.906 (0.854, 0.958)	0.493	0.129 (0.097, 0.160)	0.115 (0.086, 0.144)	0.238
24 month	0.927 (0.890, 0.964)	0.932 (0.892, 0.973)	0.795	0.128 (0.102, 0.153)	0.123 (0.098, 0.149)	0.649
36 month	0.910 (0.856, 0.963)	0.915 (0.861, 0.969)	0.817	0.132 (0.107, 0.157)	0.130 (0.101, 0.158)	0.847
48 month	0.906 (0.842, 0.969)	0.927 (0.876, 0.978)	0.434	0.136 (0.109, 0.163)	0.122 (0.096, 0.147)	0.178

## Data Availability

The original contributions presented in this study are included in the article. Further inquiries can be directed to the corresponding author.

## Supplementary Materials

The following supporting information can be downloaded at: https://www.mdpi.com/article/10.3390/curroncol33010035/s1, Figure S1: Image preprocessing pipeline for deep learning-based radiomics feature extraction; Figure S2: Comprehensive workflow of the deep-learning radiomics pipeline.

## Abbreviations

The following abbreviations are used in this manuscript:

PCa	Prostate cancer
BCR	Biochemical recurrence
PSA	Prostate-specific antigen
mpMRI	Multiparametric MRI
ADC	Apparent diffusion coefficient
AI	Artificial intelligence
ROI	Region of interest
PACS	Picture Archiving and Communication System
RT	Radiation therapy
HT	Hormone therapy
ADT	Androgen deprivation therapy
AUA	American Urological Association
CRPC	Castration-resistant prostate cancer
DWI	Diffusion-weighted imaging
T2WI	T2-weighted imaging
DCE	Dynamic contrast-enhanced
PI-RADS	Prostate Imaging Reporting and Data System
DSC	Dice similarity coefficient
VS	Volume similarity
HD	Hausdorff distance
PCA	Principal component analysis
ROC	Receiver operating characteristic
AUC	Area under the curve
HR	Hazard ratios
CI	Confidence interval
DCA	Decision curve analysis
ISUP	International Society of Urological Pathology
IQR	Interquartile ranges
PH	Proportional hazards
