# Supplementary material for: An AI-Based Radiomics Model Using MRI ADC Maps for Accurate Prediction of Advanced Prostate Cancer Progression
Source: Curr Oncol. 2026 Jan 8;33(1):35. doi: 10.3390/curroncol33010035 (PMC12840438; doi:10.3390/curroncol33010035)
Supplement: Supplementary file 1 [file curroncol-33-00035-s001.zip › Supplementary_Materials.pdf]

Subgroup analysis to compare the segmentation and predictive performance between peripheral and transitional zones

Since the tumor volumes in our cohort were relatively large, many lesions involved both the peripheral zone (PZ) and the transition zone (TZ). To classify these lesions, we compared the volume distribution between the two zones: a lesion was defined as primarily located in the PZ if its PZ component was larger than its TZ component, and vice versa. Based on this criterion, 148 (81.3%) lesions were classified as primarily located in the PZ, and 34 (18.7%) were primarily in the TZ.

We performed a subgroup analysis to compare the segmentation and predictive performance between these two anatomical regions:

1. Segmentation Performance: The AI-based segmentation achieved high consistency with manual reference in both zones. The median Dice Similarity Coefficient (DSC) was 0.905 for PZ lesions and 0.887 for TZ lesions, with no statistically significant difference (*P*=0.381), indicating that the model is robust to the complex background signals often found in the TZ.

2. Predictive Performance: In the test set, the radiomics model demonstrated comparable discrimination for both zones. The AUC for PZ lesions was 0.858 (95% CI: 0.702–0.985), while the AUC for TZ lesions was 0.832 (95% CI: 0.655–0.978). The difference was not statistically significant (DeLong test, *P*=0.119).

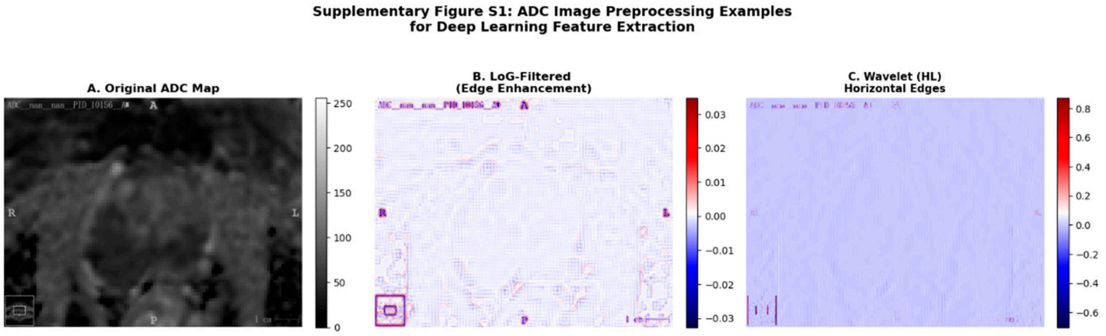

Supplementary Figure S1. Image preprocessing pipeline for deep learning-based radiomics feature extraction.

Representative examples of preprocessing steps applied to ADC maps before input to the 3D U-Net model. (A) Original ADC Map: Unprocessed ADC map from a patient with advanced prostate cancer, showing heterogeneous signal intensity in the prostate gland. The ADC map provides quantitative information about tissue cellularity and microstructure. (B) LoG-

Filtered Image (Edge Enhancement): Result of Laplacian of Gaussian (LoG) filtering ($\sigma = 2.0$) applied to the original ADC map. The LoG filter enhances edge details and texture variations, highlighting tumor boundaries and internal heterogeneity. Positive values (red) and negative values (blue) represent different edge orientations and intensity transitions, which aid in detecting subtle tumor margins and distinguishing cancerous from benign tissues. (C) Wavelet-Decomposed Image (Horizontal Edges): One component of the 2D wavelet decomposition showing horizontal detail coefficients (LH component). Wavelet decomposition using the Daubechies 1 (db1) wavelet basis extracts multi-scale texture features across different spatial frequencies. The LH component specifically captures horizontal edge information and vertical structures. Together with other wavelet components (LL, HL, HH—not shown), these preprocessed images serve as multi-channel inputs to the deep learning model, enabling the network to learn discriminative features at multiple scales for automated tumor segmentation and progression prediction. All preprocessing steps are performed automatically during model training and inference.

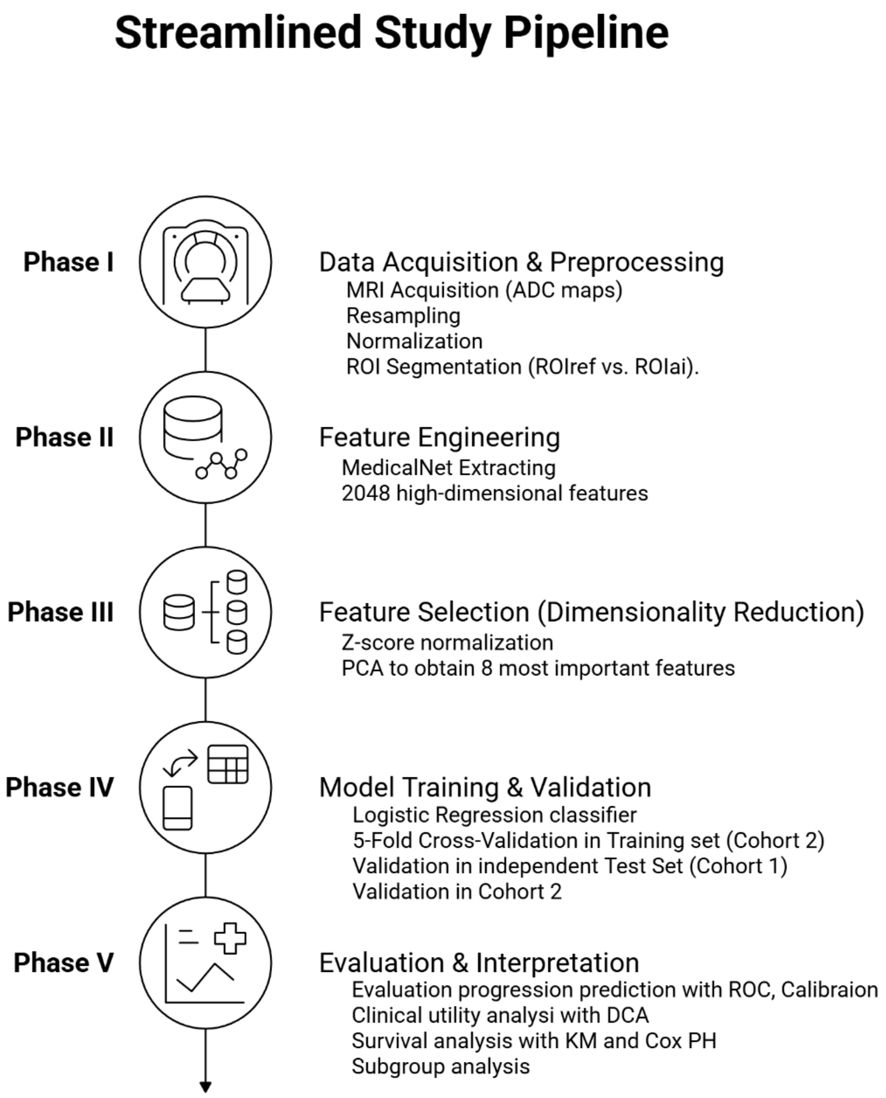

Figure S2. Comprehensive workflow of the deep-learning radiomics pipeline.

Phase I: Image Preprocessing. Pretreatment ADC maps underwent spatial resampling to a voxel size of 1 mm^3 and Z-score intensity normalization to ensure feature consistency across the cohort.

Phase II: Lesion Segmentation. Both manual and automated annotations (ROIref and ROIai) were obtained for the index lesion.

Phase III: Feature Extraction and Engineering. High-dimensional deep-learning features were extracted using the pre-trained MedicalNet–ResNet50 architecture. Extracted features were standardized using standard scaling.

Phase IV: Dimensionality Reduction and Feature Selection. Principal component analysis (PCA) with Z-score normalization was applied to identify the most robust prognostic predictors.

Phase V: Model Development and Evaluation. A logistic regression classifier was trained using 5-fold cross-validation on the training set (Cohort 1) and validated on both the independent test set (Cohort 1) and Cohort 2. Model performance was evaluated using ROC analysis. Survival analyses were performed in both Cohort 1 and Cohort 2 using Kaplan–Meier and Cox proportional hazards analyses, followed by subgroup analyses.
