# Supplementary figures and images for: An AI-Based Radiomics Model Using MRI ADC Maps for Accurate Prediction of Advanced Prostate Cancer Progression
Source: Curr Oncol. 2026 Jan 8;33(1):35. doi: 10.3390/curroncol33010035 (PMC12840438; doi:10.3390/curroncol33010035)

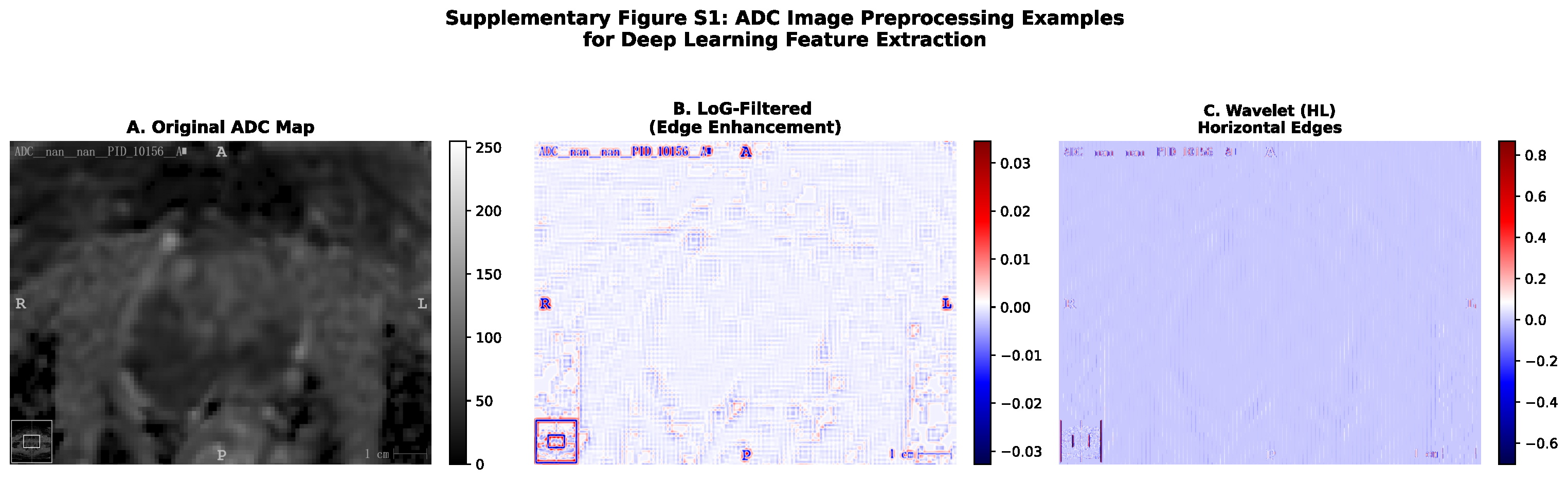

Supplement: Supplementary file 1 [file curroncol-33-00035-s001.zip › Supplementary_Figure_S1.png]

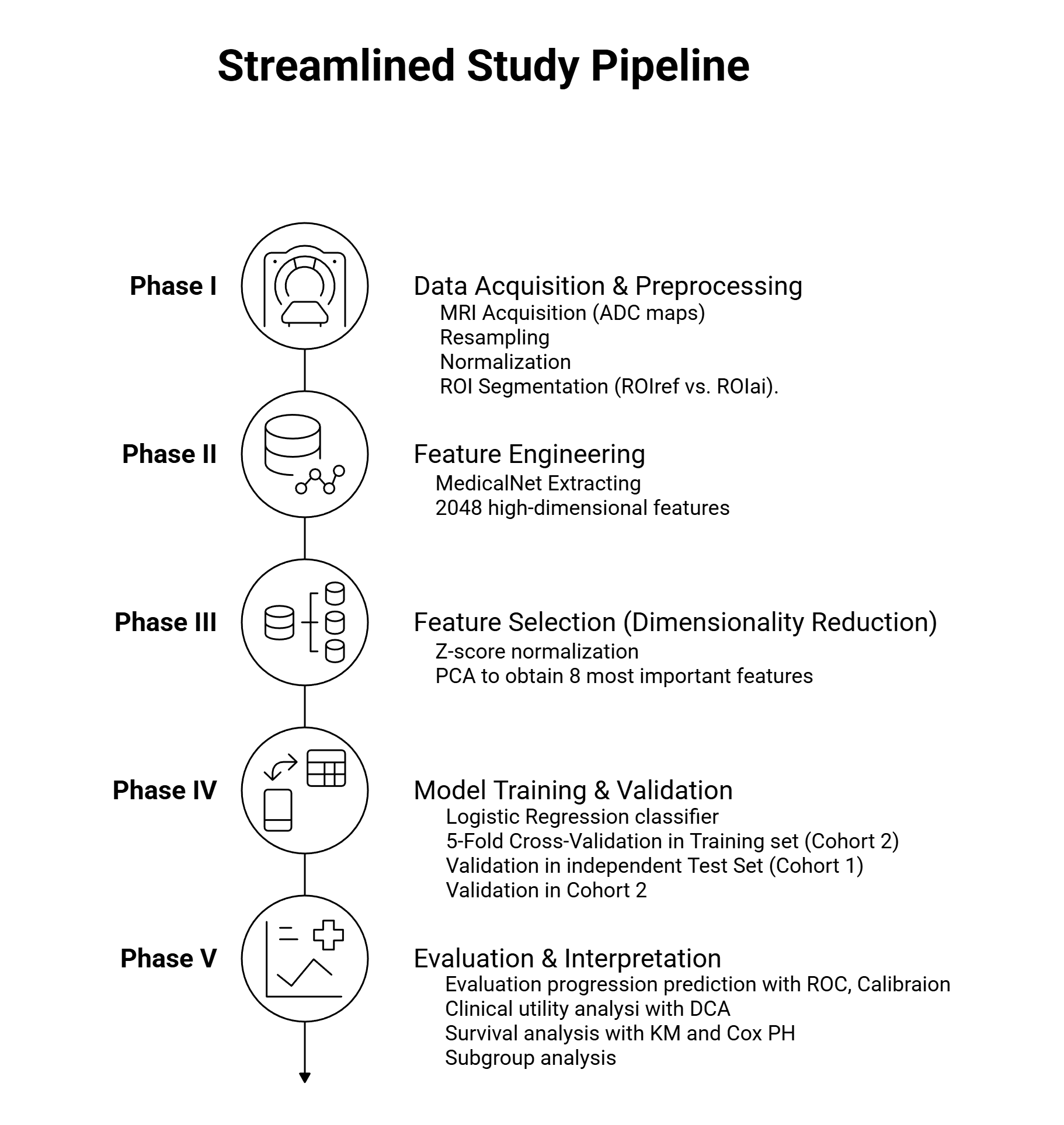

Supplement: Supplementary file 1 [file curroncol-33-00035-s001.zip › Supplementary_Figure_S2.png]
